# Effects of lifelong spontaneous exercise on skeletal muscle and angiogenesis in super-aged mice

**DOI:** 10.1371/journal.pone.0263457

**Published:** 2022-08-17

**Authors:** Kyung-Wan Baek, So-Jeong Kim, Bo-Gyu Kim, Youn-Kwan Jung, Young-Sool Hah, Hyo Youl Moon, Jun-Il Yoo, Jin Sung Park, Ji-Seok Kim

**Affiliations:** 1 Department of Physical Education, Gyeongsang National University, Jinju, Korea; 2 Research Institute of Pharmaceutical Sciences, Gyeongsang National University, Jinju, Korea; 3 Department of Convergence Medical Science, Gyeongsang National University, Jinju, Korea; 4 Biomedical Research Institute, Gyeongsang National University Hospital, Jinju, Korea; 5 Department of Physical Education, Seoul National University, Seoul, Korea; 6 Department of Orthopaedic Surgery, Gyeongsang National University Hospital, Jinju, Korea; 7 Department of Orthopaedic Surgery and Institute of Health Sciences, Gyeongsang National University School of Medicine and Gyeongsang National University Hospital, Jinju, Korea; Temple University School of Medicine, UNITED STATES

## Abstract

There has been an increasing awareness of sarcopenia, which is characterized by a concomitant decrease in skeletal muscle mass and quality due to aging. Resistance exercise is considered more effective than aerobic exercise in terms of therapeutic exercise. To confirm the effect of long-term aerobic exercise in preventing sarcopenia, we evaluated the skeletal muscle mass, quality, and angiogenic capacity of super-aged mice that had undergone lifelong spontaneous exercise (LSE) through various experiments. Our findings show that LSE could maintain skeletal muscle mass, quality, and fitness levels in super-aged mice. In addition, *ex vivo* experiments showed that the angiogenic capacity was maintained at a high level. However, these results were not consistent with the related changes in the expression of genes and/or proteins involved in protein synthesis or angiogenesis. Based on the results of previous studies, it seems certain that the expression at the molecular level does not represent the phenotypes of skeletal muscle and angiogenesis. This is because aging and long-term exercise are variables that can affect both protein synthesis and the expression patterns of angiogenesis-related genes and proteins. Therefore, in aging and exercise-related research, various physical fitness and angiogenesis variables and phenotypes should be analyzed. In conclusion, LSE appears to maintain the potential of angiogenesis and slow the aging process to maintain skeletal muscle mass and quality. Aerobic exercise may thus be effective for the prevention of sarcopenia.

## Introduction

Sarcopenia is a degenerative disease in which the mass, quality, and strength of skeletal muscles are lost due to aging [1,2]. Sarcopenia was issued a disease code (M62.84) according to the International Classification of Diseases, Tenth Revision, by the World Health Organization in 2016 [3]. The prevalence of sarcopenia is rapidly increasing with the recent global aging trend [4]. Therefore, studies are being conducted to investigate sarcopenia.

Many studies aimed at improving sarcopenia have led to the development of biological mechanisms applicable to treatment and drugs targeting them [4]. Most drugs for the treatment of sarcopenia have been developed to increase the quantity and quality of skeletal muscles. These drugs have had little effect. Furthermore, drugs that have been effective in treating sarcopenia are burdened by, side effect. For example of myostatin antibodies include urticaria, aseptic meningitis, diarrhea, confusion, and fatigue [5]; moreover, they showed no marked advantage over the combination of resistance training and protein supplementation [6]. Therefore, we can conclude that exercise and nutritional management are crucial to treating sarcopenia, except for those who cannot participate in regular practice.

After the onset of sarcopenia, resistance exercise is mainly recommended from a therapeutic point of view [7]. Resistance exercise is known to increase fiber cross-sectional area (CSA) and strength [8]. Resistance exercise is generally considered more effective than endurance exercise because it improves muscle mass loss, a fundamental problem of sarcopenia [9]. The recommended combination of resistance exercise with protein supplementation after the onset of sarcopenia is very effective [10]. In addition, resistance training and protein supplementation stimulate the mammalian target of rapamycin (mTOR) signaling pathway to promote muscle synthesis [11]. However, excessive promotion of the mTOR signaling pathway is also known to promote cellular senescence [12]. It is also known that resistance exercise and the protein anabolic response of the skeletal muscle to essential amino acids are delayed with aging [13]. Although not conclusively established, excessive resistance exercise and protein intake promote aging throughout life. At least, aerobic exercise has a more significant effect on lifespan extension [14]. For the prevention of sarcopenia, the quantity and quality of skeletal muscle are important, as is slowing of aging. Endurance exercise performed throughout life is an effective delaying aging and maintaining skeletal muscle health.

Regular endurance exercise is known to maintain energy homeostasis and delay senescence and premature death [15]. In addition, it seems necessary to consider the benefits of endurance exercise in terms of preventing sarcopenia, since endurance exercise shows a lower risk of injury and greater long-term performance potential than resistance exercise. In general, endurance exercise increases the capillary density of the skeletal muscle, but it is known that its effect in increasing the CSA of the muscle is notably lower than that of resistance exercise [8,16]. As mentioned earlier, it is undeniable that endurance exercise increases the capillary density of the muscle [16], so it can provide clear benefits to the skeletal muscle. It is unclear how vascular endothelial growth factor (VEGF) mRNA is regulated *in vivo* by changes in transcription and stability, protein synthesis and interactions with other growth factors [17]. The function, rather than the expression of VEGF, declines with aging [18]. However, aerobic exercise stimulates the induction of angiogenesis by stimulating VEGF [17]. It is believed that laminar shear stress (LSS) increased by aerobic exercise can alleviate the severity of atherosclerosis and improve vascular health [19–22]. The aging of vascular endothelial cells (ECs) is caused by factors related to vascular pathologies, such as oxidative stress (OS) [23,24]. However, it is known that aerobic exercise upregulates the expression of Sirtuins, an NAD^+^-dependent histone deacetylase, to have a positive effect on maintaining vascular function and preventing aging of ECs [25,26]. In addition, since endurance exercise is also performed through skeletal muscle fiber contraction, it can help maintain skeletal muscle mass even if muscle hypertrophy does not appear [27].

Nevertheless, the effect of endurance exercise on sarcopenia is underestimated compared to that of resistance exercise. The reason for this is that the studies so far have mainly focused on therapeutic approaches after the onset of sarcopenia. In addition, large-scale epidemiological studies are difficult to control for variables that directly or indirectly affect endurance exercise. In the case of clinical studies, it is difficult to obtain various physiological phenotypes in the muscles following endurance exercise through research because long-term endurance exercise intervention over a lifetime is virtually impossible.

Our previous study indirectly demonstrated that the risk of premature death can be lowered by maintaining energy homeostasis in super-aged mice subjected to lifelong spontaneous exercise (LSE) [15]. Therefore, it was expected that the onset of sarcopenia caused by aging would be delayed or the risk of sarcopenia occurring during aging would be lowered if LSE that mimics endurance exercise is performed.

We aimed to investigate the quantity and quality of skeletal muscle in 25-month-old super-aged mice (99-week-old, corresponding to a human age of at least > 70 years) [28], which corresponds to the late life of naturally aging mice used as a rodent model for sarcopenia [29]. In particular, one of the key points of this study was to explain capillary density (expression of angiogenesis-related genes and angiogenic capacity), which is known to be closely related to endurance exercise, by integrating the results of our previous study.

## Materials and methods

### Animal care

Experiments were performed at the Animal Care Facility of The Pusan National University School of Medicine in Yangsan, Gyeongsangnam-do. Female C57BL/6 mice (6 weeks old upon arrival) were purchased from Samtako (Daejeon, Republic of Korea). All mice were housed in a room maintained at 22–24°C, with 50–60% relative humidity, under a 12 h light/12 h dark cycle. The mice were supplied with a normal diet (AIN-93G, Dyets, Bethlehem, PA, USA) and fresh water *ad libitum*, which were replaced with fresh feed and water daily. All mice were euthanized after being deeply anesthetized with isoflurane. The intervention method in this study was not an experiment that caused pain in mice. For the welfare of the mice, new feed and drinking water were provided at least twice a week. In addition, the bedding was changed at least once a week to maintain a comfortable environment. All procedures involving animals were in accordance with the ethical standards of the Institutional Animal Care and Use and Committee of Pusan National University (approval number: PNU-2019-2448).

### Experimental design

The overall design of this study was described in our previous study [15]. After purchasing 6-week-old C57BL/6 mice for the aging study, half of the mice were bred in cages equipped with a running wheel (Amazon, Changnyeong, Republic of Korea) (Old-EXE, *n* = 7) and the remaining half were bred in cages without a wheel (Old-CON, *n* = 7). To perform the aging study, mice were maintained until 25 months of age (99 week old). For comparison with the aging study, 6-week-old young mice were bred for 16 weeks in the same manner as the mice used in the aging study (Young-CON, Young-EXE, each *n* = 7). To exclude the immediate physiological effects on the experimental animals, the spontaneous wheels were removed from the cages of the Young-EXE and Old-EXE groups 48 h before sacrifice. At the end of the aging study, young mice were simultaneously euthanized at 22 weeks of age (Young mice = 22-week-old; Old = 25-month-old).

### Physical fitness test

We reviewed the results of a physical fitness test published in our previous study to identify the level of fitness that is highly related to skeletal muscle loss and aging [15]. The data reviewed in this study were body weight, endurance test, habitual spontaneous exercise test, rotarod test, and grip strength test. The detailed protocols are described in our previous research article [15].

### Blood vessel and skeletal muscle collection

After anesthesia using isoflurane, the body weights of the experimental animals were measured on an Adventurer™ micro-analytical scale (Ohaus, Parsippany, NJ, USA). Then, the skin was incised from the lower abdomen to the sternal region, and the rib cage was opened. The full-length aorta extending 5–10 mm below the bifurcation of the iliac artery, including the subclavian right and left common carotid arteries, was removed. The excised full-length aorta was cut into the carotid and iliac arteries. The iliac artery was then quickly flushed (few seconds) using a 29-gauge insulin syringe containing QIAzol^®^ Lysis Reagent (QIAZEN Sciences, Maryland, USA) and placed in a microfuge tube. The eluate contained in the microfuge tube corresponds to ECs. The iliac artery tissue after the lumen was flushed corresponded to the smooth muscle cells (SMCs). The carotid artery was used for western blotting and aortic ring assay. After blood vessel sampling, the gastrocnemius muscle was collected. After peeling off the skin and fascia sequentially from the hind limb of the euthanized mice, the Achilles tendon was cut with scissors, and the gastrocnemius muscle was separated by lifting it with forceps. Red gastrocnemius was removed from collected gastrocnemius, and only white gastrocnemius was sampled. Muscle samples were freshly weighed on an Adventurer™ micro-analytical scale immediately after dissection.

### Western blotting

The gastrocnemius muscle, which is known to suffer the greatest loss due to aging among the skeletal muscles, was collected after sacrifice of mice and stored in a deep freezer below -80°C. The gastrocnemius muscle was used to the white gastrocnemius, which has a relatively high proportion of type II muscle fibers compared to the red gastrocnemius [30]. Frozen gastrocnemius muscles were homogenized with PRO-PREP^TM^ for Cell/Tissue (Intron Biotechnology, Seongnam, Korea). Proteins from the carotid artery were extracted the same way as the gastrocnemius and used for western blotting of angiogenesis-related proteins (VEGF and VEGFR2). The protein extracts were separated by 12% SDS-polyacrylamide gel electrophoresis and transferred to polyvinylidene difluoride (PVDF) membranes (Immobilon^®^-P, Merck Millipore, Darmstadt, Germany). The membranes were blocked in 5% (w/v) non-fat dry milk for 1 h and incubated with primary antibodies overnight at 4°C. The primary antibodies used were as follows: anti-glyceraldehyde 3-phosphate dehydrogenase (GAPDH) antibody (1:3,000, ab8245, Abcam, Cambridge, MA, USA), anti-mTOR antibody (1:1,000, ab25880, Abcam), anti-IGF1 antibody (1:1,000, ab40657, Abcam), anti- ribosomal protein S6 kinase beta-1 (S6K1) antibody (1:1,000, ab9366, Abcam), anti-beta actin antibody (1:3,000, ab8226, Abcam), anti-VEGF antibody (1:500, sc-7269, Santa Cruz Biotechnology, Santa Cruz, CA, USA) and anti-VEGFR2 antibody (1:1000, #2479, Cell Signaling Technology, Danvers, MA, USA). The membranes were then incubated for 1 h at room temperature with horseradish peroxidase-conjugated secondary antibodies (suitable for each primary antibody): goat anti-rabbit (1:3,000, #1706515, Bio-Rad, Hercules, USA) or goat anti-mouse (1:3,000, #1706516, Bio-Rad) secondary antibodies.

### Fiber cross-sectional area

Hematoxylin & eosin staining was performed to determine the fiber CSA. A paraffin-embedded block was fabricated using the gastrocnemius muscle fixed with 4% paraformaldehyde. The paraffin-embedded block was cut into slices with a thickness of 5 μm using a microtome, and the sections were placed on a glass slide. Paraffin-embedded sections were deparaffinized, hydrated, and stained with hematoxylin for 5 min. The sections were rinsed with tap water for 10 min. The sections were then decolorized using 1% HCl, rinsed with tap water for 10 min, stained with eosin for 1 min, and dehydrated in gradient alcohol. The sections were then cleared in xylene prior to sealing with neutral gum. For CSA analysis, 20 non-overlapping representative fields of the muscle tissue of each mouse were imaged under a light microscope using a 20 × magnification (Nikon, ECLIPSE Ni, Tokyo, Japan), and the CSA was measured using image analysis software (ImageJ, National Institutes of Health, Bethesda, MD, USA).

### Quantitative real-time RT-PCR (qPCR)

After sacrificing the mice, ECs and SMCs stored in a deep freezer at -80°C were homogenized. All samples were homogenized using an ultrasonicator (VCX-400, Vibra Cell, Sonics & Materials, Inc., Danbury, CT, USA). Total RNA was isolated using TRIzol reagent (Ambion, Carlsbad, CA, USA), and cDNA was synthesized from the RNA using AccuPower^®^ RT Premix (Bioneer, Daejeon, Korea) according to the manufacturer’s instructions. The cDNA was mixed with forward and reverse primers for qPCR in a 384-well plate (MicroAmp® 384-Well Reaction Plate with Barcode, Applied Biosystems, Foster City, CA, USA) containing Power SYBR® Green PCR Master Mix (Applied Biosystems, Warrington, UK).

*Pecam1* and *Acta2* were selected to differentiate between EC (*Pecam1* expression *> Acta2* expression) and SMC (*Acta2* expression *> Pecam1* expression). In addition, angiogenesis-related genes were analyzed in ECs and SMCs in triplicate. To normalize the mRNA levels among samples, *Gapdh* was amplified by qPCR as a housekeeping gene. The reaction was performed using the ViiA 7 real-time PCR system (Applied Biosystems). Primer sequences used in the experiments are listed in S1 Table. The output cycle threshold (Ct) values were then quantified [31].

### Aortic ring assay

To determine the angiogenic capacity, an *ex vivo* aortic ring assay was performed using the carotid artery w [32]. The carotid artery was collected after sacrificing the mice and immediately placed in a sample tube containing a cell growth medium (EGM^TM^-2 Endothelial Cell Growth Medium-2 BulletKit^TM^, #CC-3162, Lonza, Basel, Switzerland) pre-warmed in a water bath at 37°C. The carotid artery in the sample tube was divided into six equal rings (thickness ± 15 μm) in a petri dish. Fifteen minutes before the carotid artery ring was divided, Matrigel^®^ Matrix (Corning, Bedford, MA, USA) was dispensed on the bottom of the 24-well plate and placed in an incubator (37°C) to allow polymerization. The prepared carotid artery ring was implanted in the Matrigel^®^ Matrix (Corning, USA) to form an annular shape when viewed from above. Finally, cell growth media were added to each well and cultured in an incubator (37°C, 5% CO_2_). After one week of culture, the number of branches protruding from the aortic ring was counted using a cell imaging system (EVOS M5000, Thermo Fisher Scientific, Carlsbad, CA, USA).

### Scratch wound-healing assay

To indirectly determine the effect of senescence and aerobic exercise on the regenerative capacity of blood vessels, scratch wound-healing assays were performed after applying LSS and OS to human umbilical vein ECs (HUVECs; #CRL-1730; ATCC, Manassas, VA, USA). HUVECs were maintained in a humidified atmosphere (37°C, 5% CO_2_) in cell growth media (Lonza, Switzerland). The HUVECs were fed every 2–3 days and sub-cultured once they reached 70–80% confluence. HUVECs passaged up to nine times were isolated from the cell culture dish with trypsin. The isolated HUVECs were plated in a cell culture dish (3 × 10^6^ cells) for the wound-healing assay. Out of a total of 15 cell culture dishes, 5 dishes were subjected to LSS on a shaker (20 dyne/cm^2^), 5 dishes were treated with H_2_O_2_ (100 μM/L) to induce OS, and 5 dishes were subjected to LSS and treated with H_2_O_2_ simultaneously; the rest of the dishes were used as controls. LSS was treated on a shaker for 24 h, then H_2_O_2_ was added, and the wound-healing assay was performed 1 h later. A single scratch was made on the endothelial monolayer of the cell culture dish using a micropipette tip. Subsequently, the cells were washed once with phosphate-buffered saline and cultured under the same conditions. The surface of all scratches (10 Ⅹ magnification) was observed using a cell imaging system (EVOS M5000, Thermo Fisher Scientific) at 0 h and 9 h of culture. Migratory distance was measured using the ImageJ program (National Institutes of Health, USA).

### Tube formation assay

A tube formation assay was performed to indirectly determine the effect of aging and aerobic exercise on angiogenic capacity. All culture conditions were the same as those used in the wound healing assay. In addition, LSS application and H_2_O_2_ treatment conditions were the same as those used in the wound-healing assay. After sub-culturing the HUVECs up to 9 times, cells in cell growth medium were dispensed in a 24-well plate with Matrigel^®^ Matrix (Corning, USA). After 9 h, cell tube formation (10 Ⅹ magnification) was captured using a cell imaging system (EVOS M5000, Thermo Fisher Scientific). The number of branch points, total tube lengths, and tube formation areas were measured [33] in the captured images using the ImageJ software (National Institutes of Health).

### Statistical analyses

The results obtained by the physical fitness test, western blotting, qPCR, and *ex vivo* aortic ring assay were statistically analyzed by two-way ANOVA and Sidak’s *post hoc* test. The results of the scratch wound-healing assay and tube formation assay were statistically analyzed by one-way ANOVA Tukey’s *post hoc* test. All statistical analyses were performed using the GraphPad Prism ver. 8 (GraphPad Software, La Jolla, CA, USA). Statistical significance was set at *p* < 0.05.

## Results

### Effects of maintaining physical fitness through LSE

**Fig 1:**
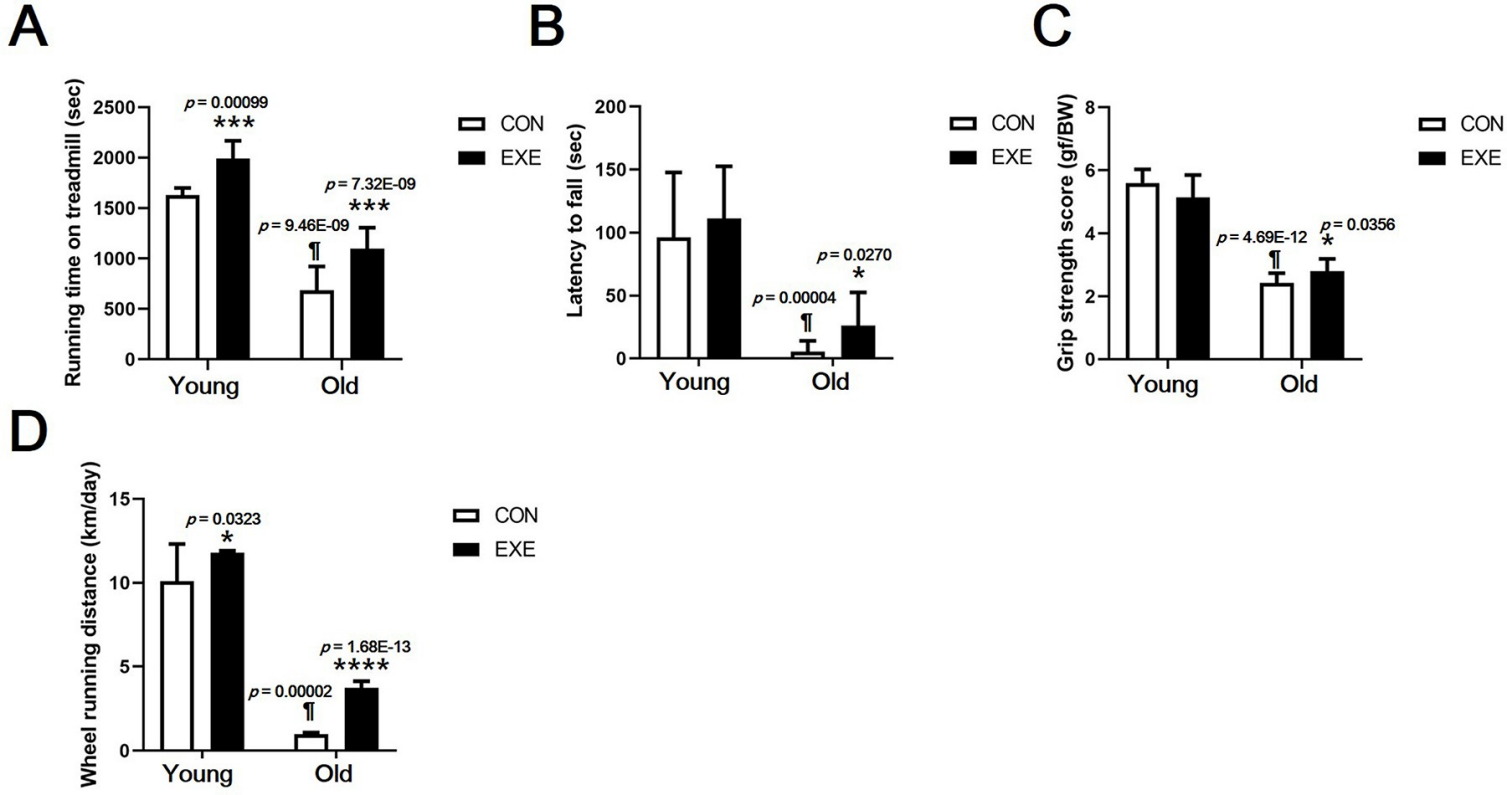
Lifelong spontaneous exercise (LSE) delays the decline in physical fitness attributed to aging. (A) Endurance test. (B) Rotarod test. (C) Grip strength test. (D) Habitual spontaneous exercise test. All data are presented as the mean ± standard deviation (SD); vs. control group of same age, *p < 0.05 and ***p < 0.001; vs. control group of different age, ^¶^*p* < 0.0001. Partially modified from Fig 3 of a previous study [15].

We reaffirmed the physical fitness test results of our previous study [15]. In the treadmill test conducted to determine endurance, the time required for exhaustion to set in was significantly shorter in Old-CON than in Young-CON mice (*p* < 0.0001), significantly longer in Young-EXE than in Young-CON mice (*p* < 0.001), and significantly longer in Old-EXE than in Old-CON mice (*p* < 0.001) (Fig 1A). In the rotarod test, the time spent on the rod was significantly shorter in Old-CON than in Young-CON mice (*p* < 0.0001); however, the Old-EXE group spent a longer time on the rotarod than the Old-CON group (*p* < 0.05) (Fig 1B). In the grip strength test, Old-CON mice demonstrated a lower grip strength score than the Young-CON mice (*p* < 0.0001), and Old-EXE mice had a higher score than the Old-CON mice (*p* < 0.05**)** (Fig 1C). The spontaneous running distance confirmed that the Old-CON mice ran a significantly shorter distance than the Young-CON (*p* < 0.0001). Additionally, both the Young-EXE (*p* < 0.05) and Old-EXE (*p* < 0.0001) groups demonstrated significantly longer spontaneous running distances than the control groups of the same age (Fig 3D).

### Effect of LSE on skeletal muscle mass

**Fig 2:**
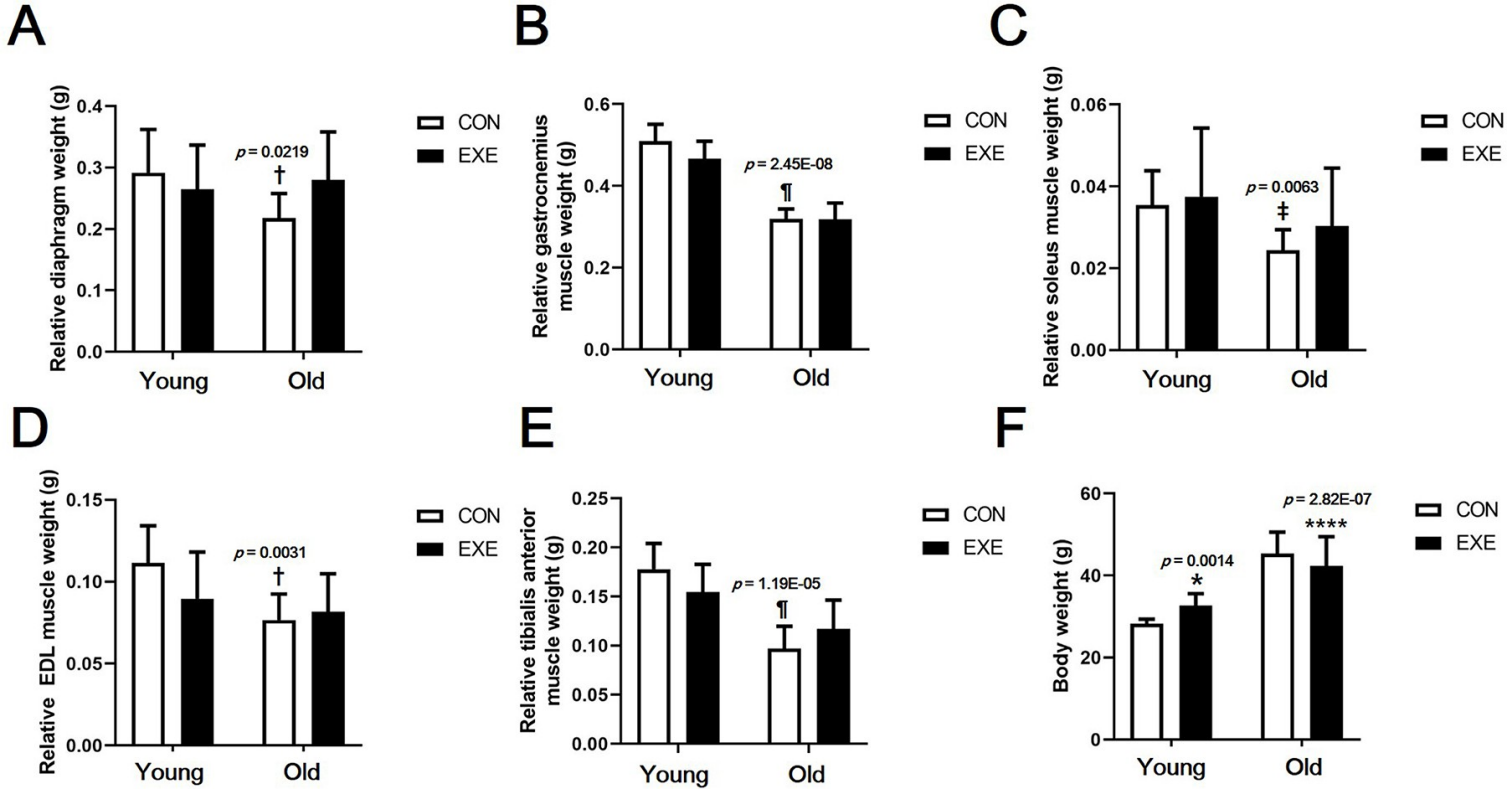
Difference in the mass of various skeletal muscles of experimental mice subjected to LSE. (A) Relative diaphragm weight. (B) Relative gastrocnemius muscle weight. (C) Relative soleus muscle weight. (D) Relative extensor digitorum longus muscle weight. (E) Relative tibialis anterior muscle weight. (F) Body weight. All data are presented as the mean ± SD vs. control group of same age, *p < 0.05 and ***p < 0.001; vs. control group of different age, ^†^*p* < 0.05, ^‡^*p* < 0.01, and ^¶^*p* < 0.0001.

To confirm the effect of LSE on sarcopenia, which was the ultimate purpose of this study, the weight of most skeletal muscles in experimental mice was measured. The weight of all skeletal muscles (diaphragm, *p* < 0.05; gastrocnemius muscle, *p* < 0.001; soleus muscle, *p* < 0.01; extensor digitorum longus (EDL) muscle, *p* < 0.01; tibialis anterior muscle, *p* < 0.001) was significantly lower in the Old-CON than in the Young-CON group (Fig 2). Body weight was significantly higher in Young-EXE than in Young-CON mice (*p* < 0.05) but significantly lower in Old-EXE than in Old-CON mice (*p* < 0.0001) (Fig 2F). All skeletal muscle weights were relative to body weight.

### Fiber CSA

**Fig 3:**
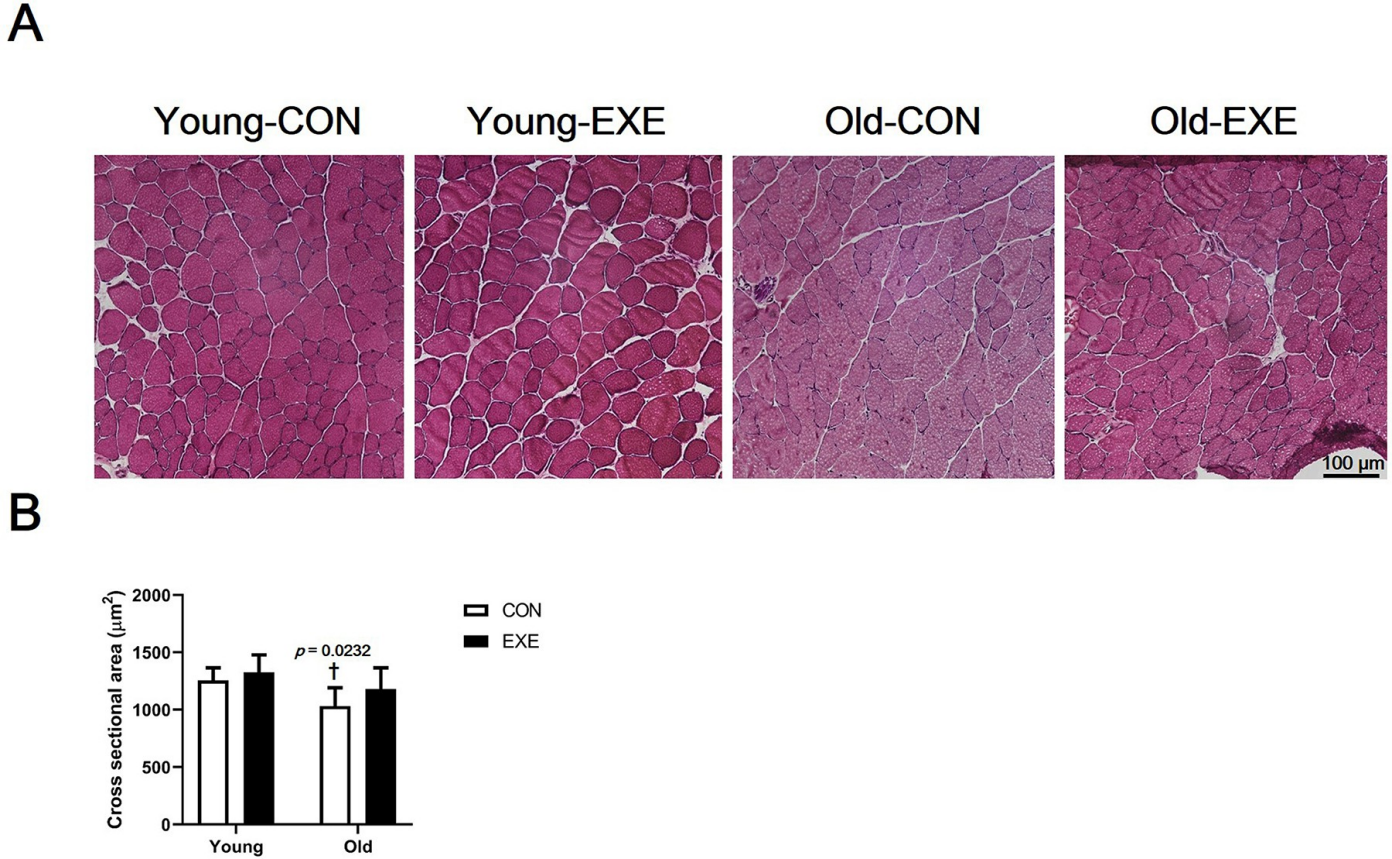
Difference in fiber-cross sectional area (CSA) of skeletal muscles of experimental mice subjected to LSE. (A) Skeletal muscle tissue microscope slides stained with Hematoxylin & Eosin. (B) Comparison of skeletal muscle fiber CSA between groups. All data are presented as the mean ± SD vs. control group of different age, ^†^*p* < 0.05.

The fiber CSA of gastrocnemius muscle fibers was significantly narrower in Old-CON than in Young-CON (*p* < 0.05). There was no significant difference in cross-sectional area between the other groups (Fig 3).

### Expression of skeletal muscle synthesis-related proteins

**Fig 4:**
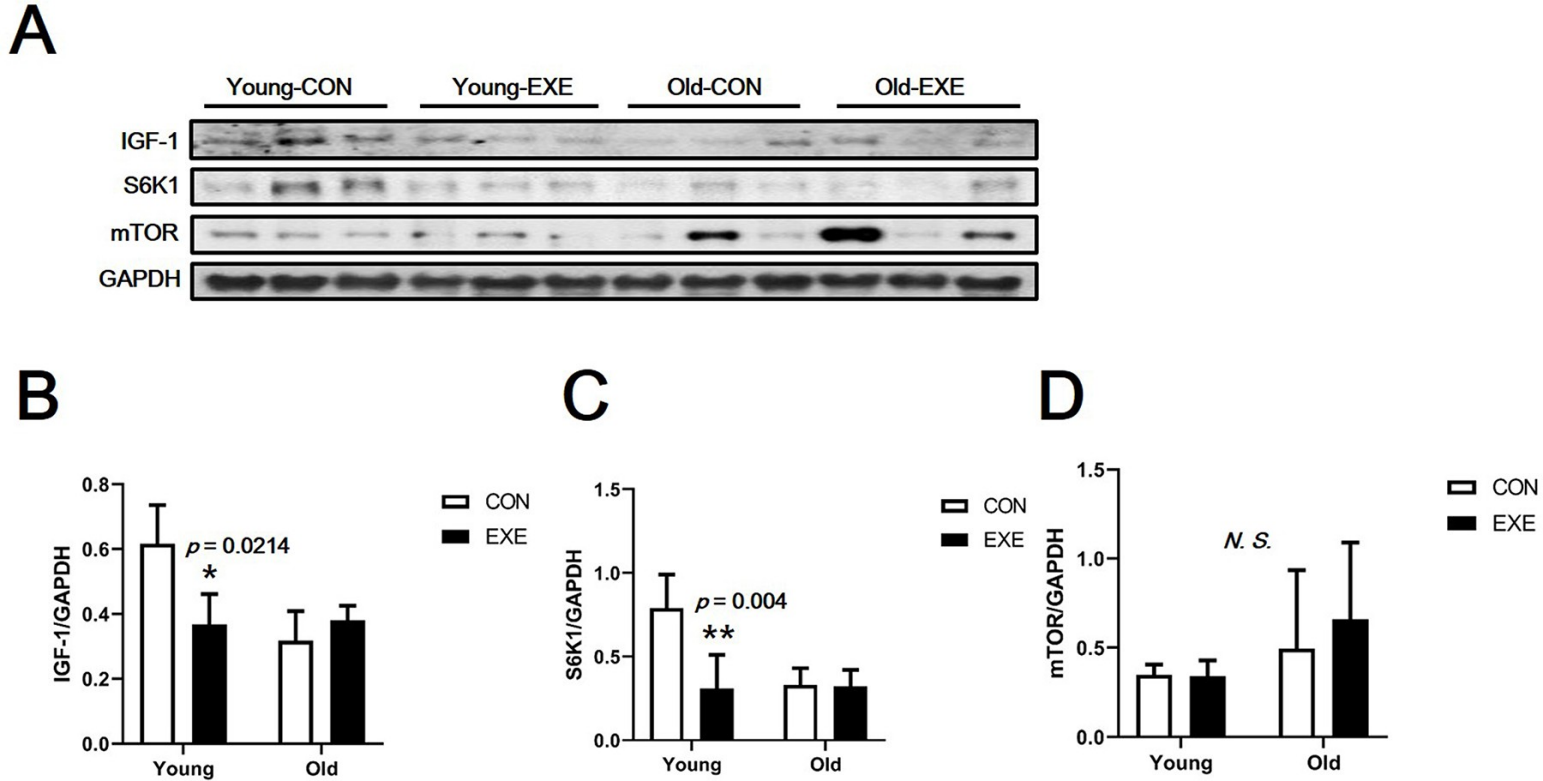
Expression of skeletal muscle synthesis-related proteins in mice subjected to LSE. (A) Western blotting band images (results of skeletal muscle synthesis-related protein analysis). (B) IGF-1 expression level. (C) S6K1 expression level. (D) mTOR expression level. All data are presented as the mean ± SD vs. control group of same age, ^†^*p* < 0.05 and ^‡^*p* < 0.01.

The expression of insulin like growth factor 1 (IGF-1) and S6K1 was significantly lower in Young-EXE than in Young-CON mice (IGF-1, *p* < 0.05; S6K1, *p* < 0.01), and there was no significant difference between the other groups (Fig 4B and 4C). The expression of mTOR was not significantly different between the groups (Fig 4D).

### Effect of LSE on angiogenesis-related gene expression and protein expression

**Fig 5:**
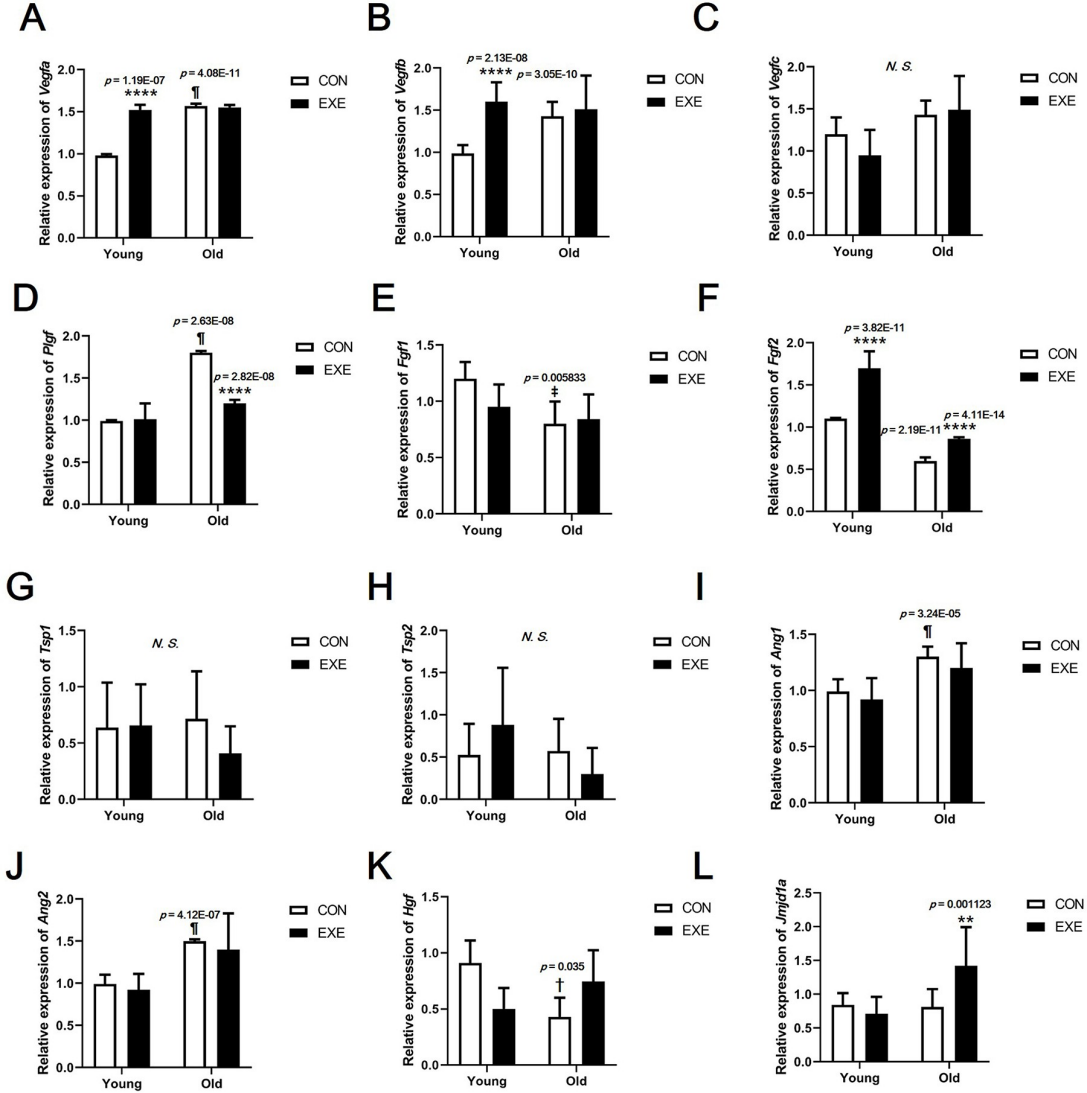
Expression of angiogenesis-related genes in endothelial cells (ECs) isolated from blood vessels of mice subjected to LSE. (A) *Vegfa* expression level. (B) *Vegfb* expression level. (C) *Vegfc* expression level. (D) *Plgf* expression level. (E) *Fgf1* expression level. (F) *Fgf2* expression level. (G) *Tsp1* expression level. (H) *Tsp2* expression level. (I) *Ang1* expression level. (J) *Ang2* expression level. (K) *Hgf* expression level. (L) *Jmjd1a* expression level. All data are presented as the mean ± SD vs. control group of same age, **p < 0.01 and ****p < 0.0001; vs. control group of different age, ^†^*p* < 0.05, ^‡^*p* < 0.01, and ^¶^*p* < 0.0001. *N*. *S*. = no significant difference between groups.

In EC, *Pecam1* expression was significantly higher than *Acta2* (*p* < 0.001). In SMC, *Acta2* expression was significantly higher than *Pecam1* expression (*p* < 0.001) (S1 Fig). Therefore, we confirmed that the ECs and SMCs were clearly isolated. In EC, *Vegfa* and *Vegfb* expression was significantly higher in Young-EXE than in Young-CON mice (*p* < 0.0001) and in Old-CON than in Young-CON mice (*Vegfa*, *p* < 0.001; *Vegfb*, *p* < 0.0001) (Fig 5A and 5B). *Vegfc* levels were not significantly different between all the groups (Fig 5C). *Plgf* expression was significantly higher in Old-CON than in Young-CON mice (*p* < 0.0001) and significantly lower in Old-EXE than in Old-CON mice (*p* < 0.0001) (Fig 5D). *Fgf1* expression was significantly lower in Old-CON than in Young-CON mice (*p* < 0.01) (Fig 5E). *Fgf2* expression was lower in Old-CON than in Young-CON mice (*p* < 0.001) and significantly higher in the exercise group (Young-EXE, *p* < 0.0001; Old-EXE, *p* < 0.0001) than in the control group of the same age (Fig 5F). *Tsp1* and *Tsp2* expression was not significantly different between the groups (Fig 5G). *Ang1* and *Ang2* expression was significantly higher in Old-CON than in Young-CON mice (*Ang1*, *p* < 0.001; *Ang2*, *p* < 0.0001) (Fig 5I and 5J). *Hgf* expression was significantly lower in Old-CON than in Young-CON mice (*p* < 0.05) (Fig 5K). *Jmjd1a* expression was significantly higher in Old-EXE than in Old-CON mice (*p* < 0.01) (Fig 5L). In the SMC, the expression of angiogenesis-related genes did not show a significant difference between the groups (S2 Fig).

### Effect of LSE on angiogenesis-related protein expression

**Fig 6:**
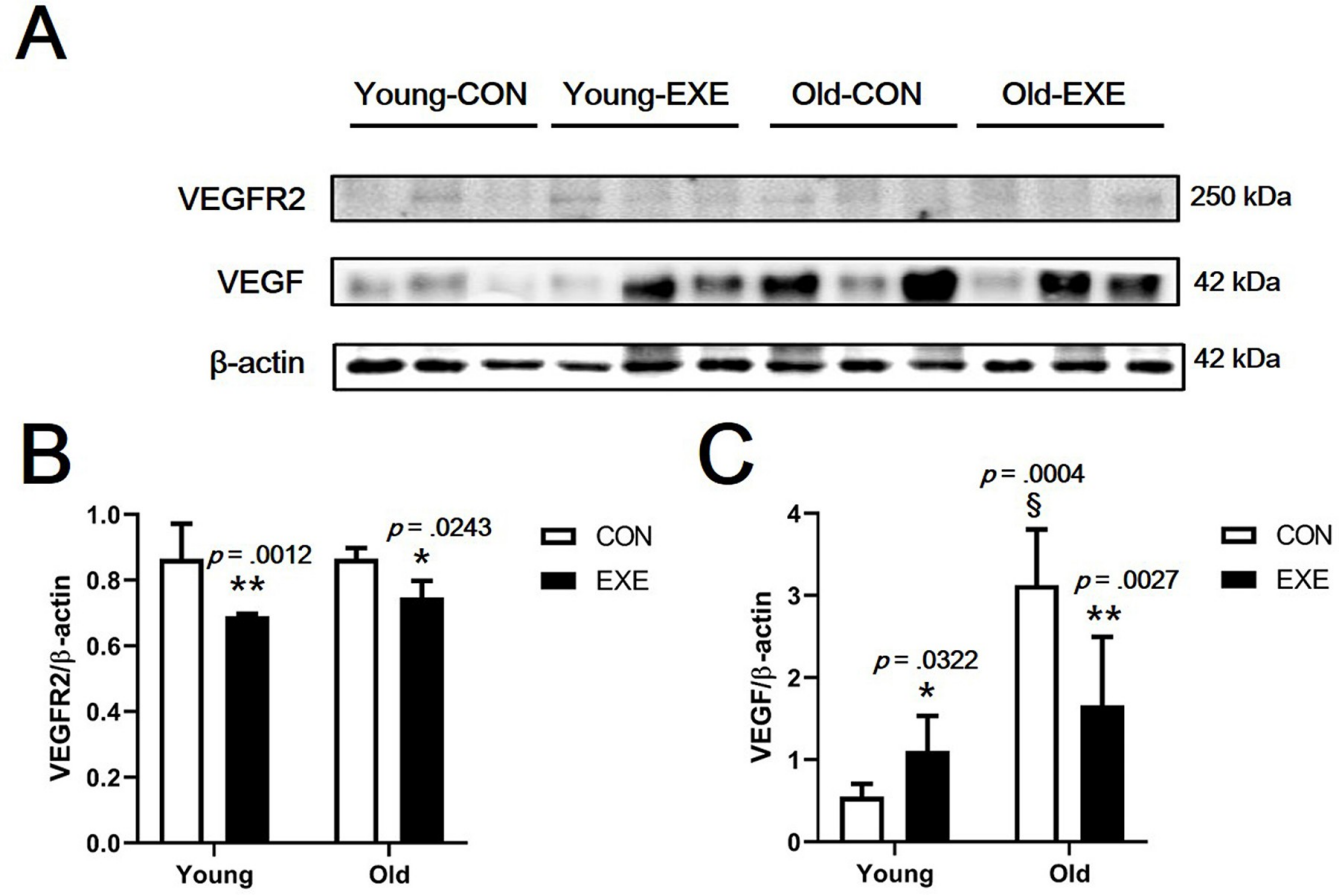
Expression of angiogenesis-related proteins in blood vessels of mice subjected to LSE. (A) Western blotting band images (results of angiogenesis-related proteins in carotid artery). (B) VEGFR2 expression level. (C) VEGF expression level. All data are presented as the mean ± SD vs. control group of same age, *p < 0.05 and **p < 0.01; vs. control group of different age, ^§^*p* < 0.001.

We confirmed the expression of VEGF, the most representative angiogenesis-related protein, and the expression of its receptor protein, vascular endothelial growth factor receptor 2 (VEGFR2), by western blotting (Fig 6A). VEGF expression was significantly higher in Young-EXE than in Young-CON mice (*p* < 0.05) and significantly lower in Old-EXE than in Old-CON mice (*p* < 0.01). In addition, the VEGF expression in Old-CON was significantly higher than that in Young-CON mice (*p* < 0.001) (Fig 6C). VEGFR2 expression was significantly lower in Young-EXE than in Young-CON mice (*p* < 0.05) and in Old-EXE than in Old-CON mice (*p* < 0.01) (Fig 6B).

### Confirmation of the effect of LSE on angiogenic capacity via *ex vivo* and *in vitro* study

**Fig 7:**
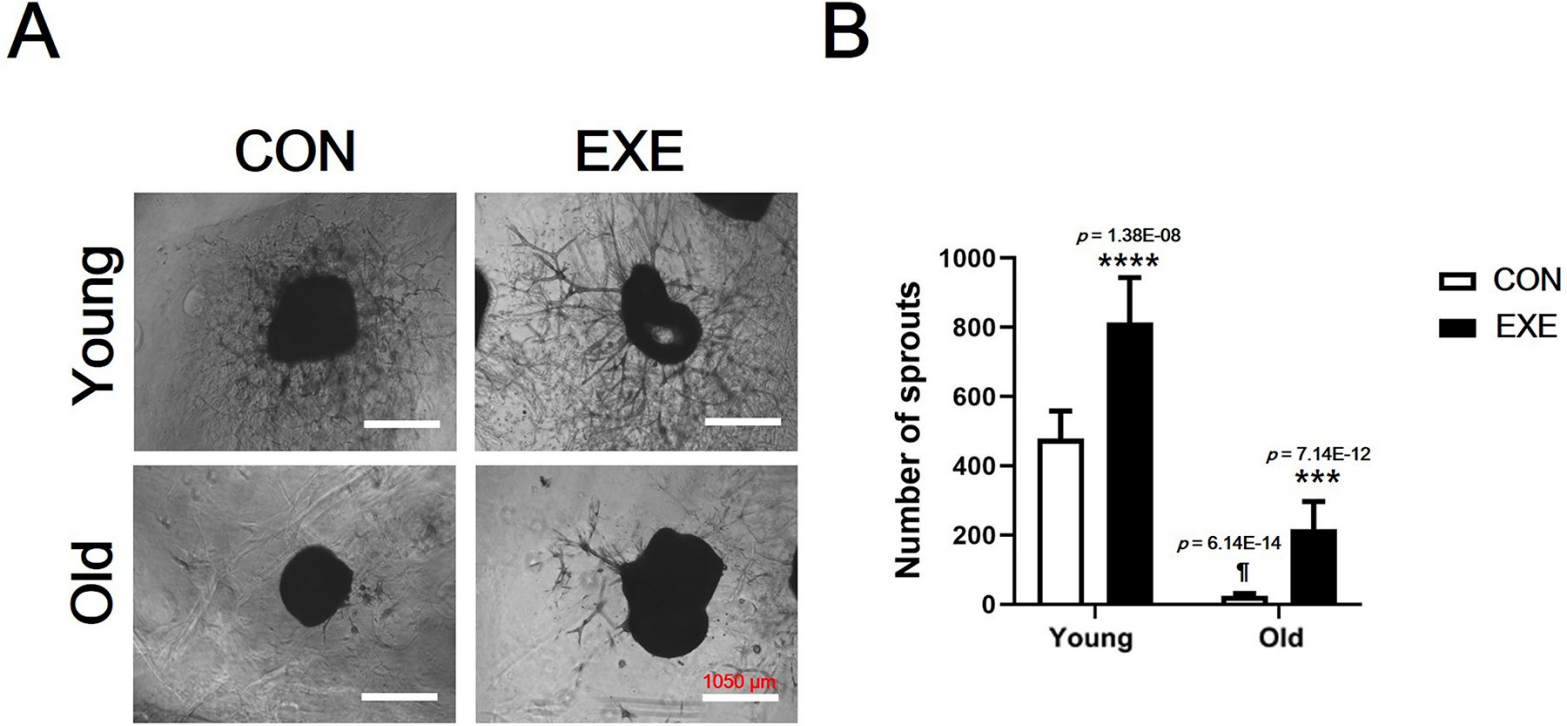
Inhibitory effect of LSE on the reduction of angiogenic capacity confirmed by *ex vivo* aortic ring assay. (A) 10Ⅹ magnification microscopic images of aortic ring assay. (B) Comparison of the number of sprouts extending after culture of isolated mouse aorta. All data are presented as the mean ± SD vs. control group of same age, ***p < 0.001 and ****p < 0.0001; vs. control group of different age, ^¶^*p* < 0.0001.

**Fig 8:**
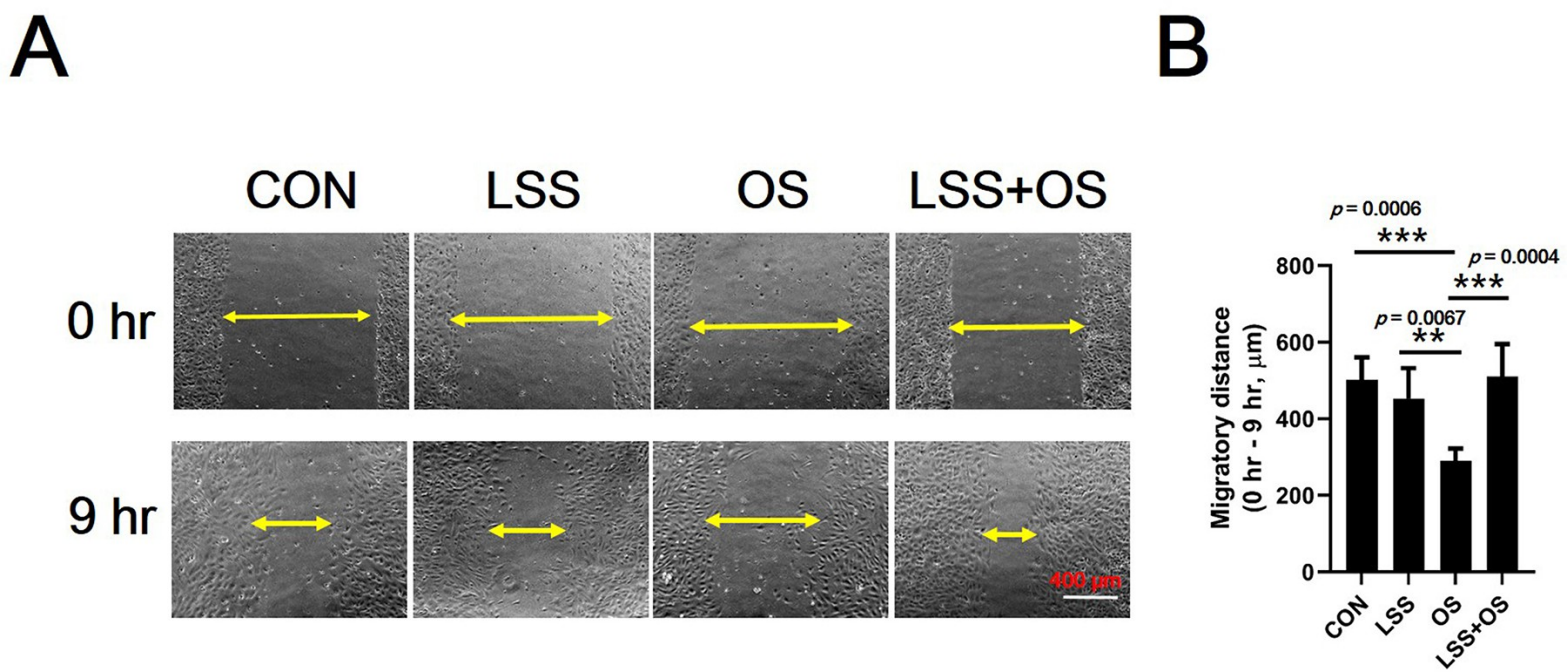
Comparison of wound healing ability of vascular ECs after senescence simulated by oxidative stress and exercise simulated by laminar shear stress *in vitro*. (A) 10Ⅹ magnification microscopic images of scratch wound healing assay. **(**B) Migratory distance. LSS: Laminar shear stress; OS: Oxidative stress. All data are presented as the mean ± SD, *p < 0.01 and ***p < 0.001.

**Fig 9:**
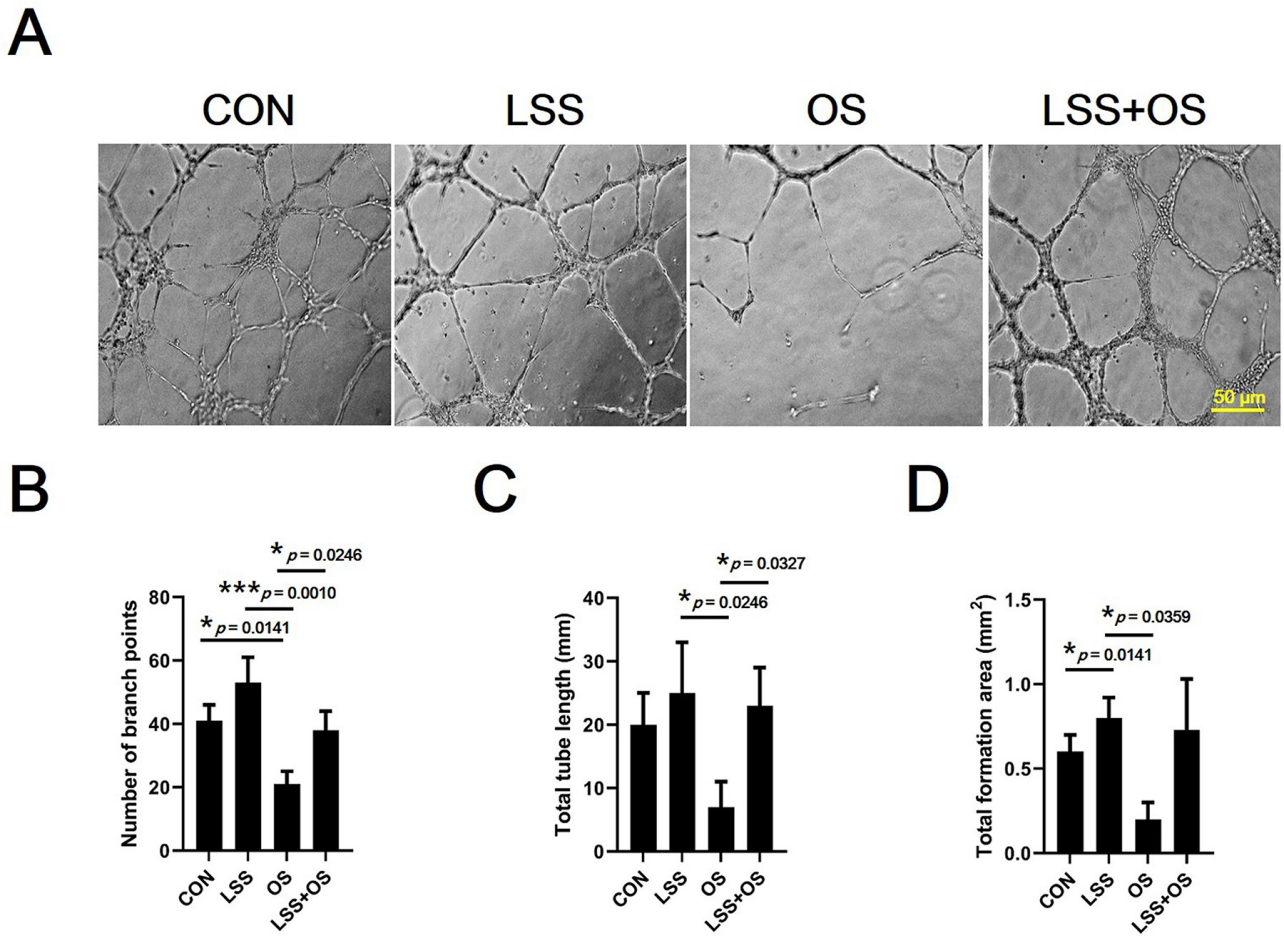
Comparison of tube formation ability of vascular ECs after senescence simulated by oxidative stress and exercise simulated by laminar shear stress *in vitro*. (A) 10Ⅹ magnification microscopic images of tube formation assay. (B) Number of branch points. (C) Total tube length. (C) Total formation area. LSS: Laminar shear stress; OS: Oxidative stress. All data are presented as the mean ± SD, *p < 0.01 and ***p < 0.001.

The results of the angiogenic capacity of blood vessels obtained from experimental mice were confirmed by an *ex vivo* aortic ring assay (Fig 7A). The Old-CON group was confirmed to have a significantly lower angiogenic capacity (number of sprouts) than the Young-CON group (*p* < 0.0001). In addition, it was confirmed that the exercise group (Young EXE, *p* < 0.0001; Old-EXE, *p* < 0.001) had higher angiogenic capacity than the control group of the same age (Fig 7B). To verify the angiogenic capacity shown by the *ex vivo* aortic ring assay *in vitro*, a scratch wound-healing assay and tube formation assay were performed (Figs 8 and 9). The wound-healing ability of vascular ECs was significantly lower in OS conditions compared to the control conditions (*p* < 0.001), LSS conditions (*p* < 0.01) and LSS+OS conditions (*p* < 0.001) (Fig 8). According to the results of the tube formation assay (Fig 9), the number of branch points was significantly lower in OS conditions compared to the control conditions (*p* < 0.05) (Fig 9B). All items (number of branch points, total tube length, and total formation area) were significantly lower in OS conditions than in LSS conditions (number of branch points, *p* < 0.001; total tube length, *p* < 0.05; total tuber formation, *p* < 0.05) and were significantly higher in LSS+OS conditions than in OS conditions (number of branch points, *p* < 0.05; total tube length, *p* < 0.05; total tube formation, *p* < 0.05) (Fig 9B–9D).

## Discussion

### The value of aerobic exercise on the prevention of sarcopenia

Our present study demonstrates that lifelong spontaneous wheel running in experimental animals mimicking sustained human aerobic exercise can help alleviate the overall sarcopenia. It was shown that the decrease in skeletal muscle of the whole body due to aging can be largely offset by LSE. This was confirmed by the grip strength (Fig 1C), relative muscle weight (Fig 2), and CSA of skeletal muscle fiber data (Fig 3). The ultimate purpose of this study was to examine the sarcopenia-prevention effect of lifelong aerobic exercise. Our results show that lifelong aerobic exercise is effective in preventing loss of muscle mass and quality. It has been shown that aerobic exercise increases muscle mass, strength, and mitochondrial biosynthesis and fission proteins in older subjects [34,35]. The results of this research prove the effectiveness of aerobic exercise as a treatment method for sarcopenia. This is because exercise intervention was performed after aging had progressed, and thus, the data do not prove the preventive effect of aerobic exercise on sarcopenia. In addition, if the effects of aerobic exercise and resistance exercise are compared after aging or onset of sarcopenia, resistance exercise has much better effects on muscle hypertrophy and muscle strength improvement. Therefore, resistance exercise is suggested as the primary treatment for sarcopenia [5]. It is widely known that resistance exercise is superior to aerobic exercise and its value as a therapeutic intervention. However, relatively low-intensity aerobic exercise can be performed for a lifetime. It may be possible to delay the onset of sarcopenia because it can delay aging. This is also found in the content related to the mTOR signaling pathway, which will be described later in the next paragraph.

### Effects of LSE and aging on the mTOR signaling pathway

In our study, which confirmed that the mTOR signaling pathway is involved in both aging and protein synthesis, aerobic exercise in aged mice caused no difference in the expression of all the related proteins (Fig 4). However, mTOR expression tended to be higher in aged mice than in young mice, but the difference was not significant. Although there was no significant difference, mTOR expression is known to increase with aging; thus, this is a worthwhile reference [36–38]. Notably, the protein expression levels of IGF-1 and S6K1 were lower in Young-EXE than in Young-CON mice (Fig 4). These may be because, due to the characteristics of voluntary wheel running that simulates aerobic exercise, excessive load was not given to the skeletal muscles of the experimental animals as much as resistance exercise. In other words, it is thought that a pathway antagonistic to mTOR was stimulated by resistance exercise due to stimulation by aerobic exercise. In any case, it is clear that the protein synthesis pathway is not stimulated by LSE in aged mice, and the response to aerobic exercise appears to be clearly different in young and aged mice. In addition, these data may be the result of the complex role of both age and exercise period in mice. However, in the present study, protein expression analysis of mouse skeletal muscle after stimulation with a single bout of exercise was not performed. Thus, it was not possible to clearly identify the difference in the expression of muscle synthesis protein or protein synthesis capacity after stimulation by exercise. The timing of the evaluation of muscle synthesis proteins is important [39]; this may be a limitation of this study. In future studies, it would be beneficial to focus on a specific protein related to muscle synthesis and evaluate it at an appropriate analysis time after exercise stimulation (e. g., puromycin assay). Other analytical methods, such as activity measurements of satellite cells, are also worth considering.

### Effects of LSE and aging on the angiogenesis

Expression levels of most of the angiogenesis-related genes analyzed in vascular ECs were found to be higher in Old-CON than in Young-CON (*Vegfa*, *Vegfb*, *Plgf*, *Ang1*, and *Ang2*) (Fig 5). These results showed that the expression levels of many genes among the anigiogenesis-related genes increased with aging. In the case of *Vegfa* and *Vegfb*, there was no significant difference between Old-CON and Old-EXE (Fig 5A and 5B). In addition, among the genes for which a significant expression difference was confirmed between Young-CON and Old-CON, the genes for which a significant expression difference between Old-CON and Old-EXE were confirmed were *Plgf* and *Fgf2* (Fig 5D and 5F). If only the gene expression level was evaluated, the effect of LSE on *Vegfa* and *Vegfb* can be interpreted as insignificant. However, there are research results that VEGF is the target of a negative feedback mechanism as exercise adaptation occurs [40]. Therefore, it appears that adaptation by LSE is highly likely to have affected the expression of various angiogenesis-related genes. In addition, since the expression of *Plgf* and *Fgf2* is not likely to directly stimulate angiogenesis by exercise, the results of gene expression analysis do not completely represent the angiogenic capacity [41]. We believe that an in-depth discussion of these issues is necessary. To this end, we searched for studies related to VEGF that were evaluated in terms of gene expression and protein expression in our study (Fig 6). The results of this study may not be easily understood because it can be taken for granted that angiogenesis decreases with aging and, therefore, the expression of related genes and proteins decreases. However, there are several clues to understanding these results. In a study examining the delay of angiogenesis due to aging, a polyvinyl alcohol sponge was implanted to examine angiogenesis in experimental mice of different ages (Young, 6–8 months vs. Old, 23–25 months) and same recovery period (14 days) [18]. The study showed that the expression of VEGF was lower in aged mice than in young mice at 14 days recovery time. However, when the recovery period was delayed to 19 days, the expression of VEGF and TSP-2 in aged mice was similar to that in young mice at 14 days recovery time [18]. It was suggested that after 19 days, VEGF expression in aged mice may be higher than that in young mice at 14 days recovery period. Therefore, it is possible that the VEGF expression was relatively high in the super-aged mice used in our study and could not be excluded as the recovery time after ischemic injury was delayed (a limitation of this study was that the timing of ischemic injury to blood vessels could not be determined). Another study showed that VEGFR2 expression was similar between both young and old rabbits [42]. It was also pointed out that the ultimate level of angiogenesis for ischemic injury recovery in old animals after treatment with recombinant VEGF therapy was far inferior to that observed in young animals. In conclusion, several studies have shown that differences in the expression of VEGF and VEGFR2 in tissues cannot functionally reflect differences in angiogenesis between young and old animals [42]. Therefore, it should be kept in mind that the differences in gene and protein expression shown in our study may not be the result of only age and exercise, and ultimately, an assay to confirm angiogenic capacity is required. Confirmation experiments (*ex vivo* and *in vitro*) showed that the angiogenic capacity decreased with aging and that exercise could offset the decrease in angiogenesis caused by aging. In particular, in the *ex vivo* aortic ring assay, blood vessels from experimental animals were extracted and cultured. Therefore, the aortic ring assay is the most definitive study to confirm whether the mice subjected to LSE maintain the angiogenic capacity. The aortic ring assay indicated that the angiogenic capacity of blood vessels was lower in aged mice than in young mice, but regardless of age, the exercise group had higher angiogenic capacity than the control group, suggesting that exercise maintains or protects the angiogenic capacity (Fig 7). An additional *in vitro* experiment was conducted to indirectly evaluate the angiogenesis capacity during aging and exercise. The scratch wound-healing assay and tube formation assay also showed that OS, mimicking aging, was offset when LSS, mimicking exercise, was used (OS vs. LSS+OS) (Figs 8 and 9).

This study showed that protein synthesis and angiogenesis-related genes and proteins must be analyzed together with physical fitness and various phenotypes to analyze the effect of aging and exercise. Taken together, our results show that the quantity and quality of skeletal muscles are maintained by delaying aging due to lifelong spontaneous aerobic exercise and maintaining the function of angiogenesis. These features appear to be independent of the synthesis of proteins involved in skeletal muscle growth.

## Conclusions

In conclusion, lifelong aerobic exercise is an excellent way to prevent sarcopenia by maintaining the angiogenic capacity, mass, and quality of skeletal muscles. However, aging and prolonged exercise can affect the expression of genes and proteins related to protein synthesis and angiogenesis. Therefore, in aging and exercise-related studies, it is necessary to prevent distorted interpretation through analysis of physical fitness test and various phenotypes rather than relying on results at the molecular level.

## Supporting information

S1 Fig Difference in the expression of specific genes in isolated vascular endothelial cells and smooth muscle cells. All data are presented as the mean ± SD. *Pecam1* vs. *Acta2*, ***p < 0.001.
(TIFF)

S2 Fig Expression of angiogenesis-related genes in the blood vessels of mice subjected to LSE. (A) *Vegfa* expression level. (B) *Vegfb* expression level. (C) *Vegfc* expression level. (D) *Plgf* expression level. (E) *Fgf1* expression level. (F) *Fgf2* expression level. (G) *Tsp1* expression level. (H) *Tsp2* expression level. (I) *Ang1* expression level. (J) *Ang2* expression level. (K) *Hgf* expression level. (L) *Jmjd1a* expression level. All data are presented as the mean ± SD. *N*. *S*. = no significant difference between groups.
(TIF)

S1 Table Primer sequences of target genes for quantitative real-time RT-PCR analysis. (XLSX)

S1 Raw images (PDF)
