# Supplementary figures and images for: Effects of lifelong spontaneous exercise on skeletal muscle and angiogenesis in super-aged mice
Source: PLoS One. 2022 Aug 17;17(8):e0263457. doi: 10.1371/journal.pone.0263457 (PMC9384990; doi:10.1371/journal.pone.0263457)

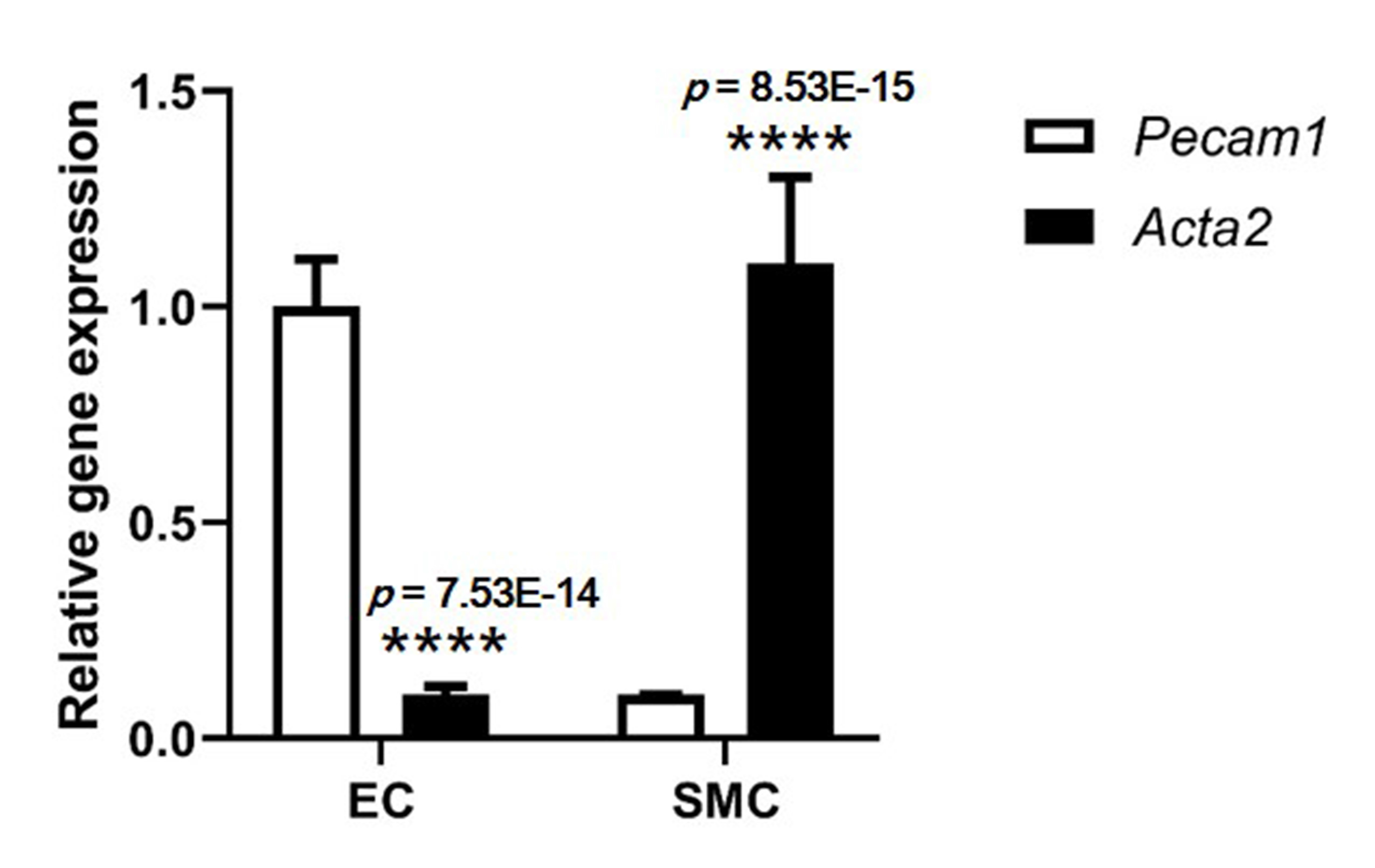

Supplement: S1 Fig — All data are presented as the mean ± SD. Pecam1 vs. Acta2, ***p < 0.001. (TIFF) [file pone.0263457.s001.tiff]
