# Supplementary material for: Effects of lifelong spontaneous exercise on skeletal muscle and angiogenesis in super-aged mice
Source: PLoS One. 2022 Aug 17;17(8):e0263457. doi: 10.1371/journal.pone.0263457 (PMC9384990; doi:10.1371/journal.pone.0263457)
Supplement: S1 Raw images — (PDF) [file pone.0263457.s004.pdf]

Young-CON Young-EXE Old-CON Old-EXE

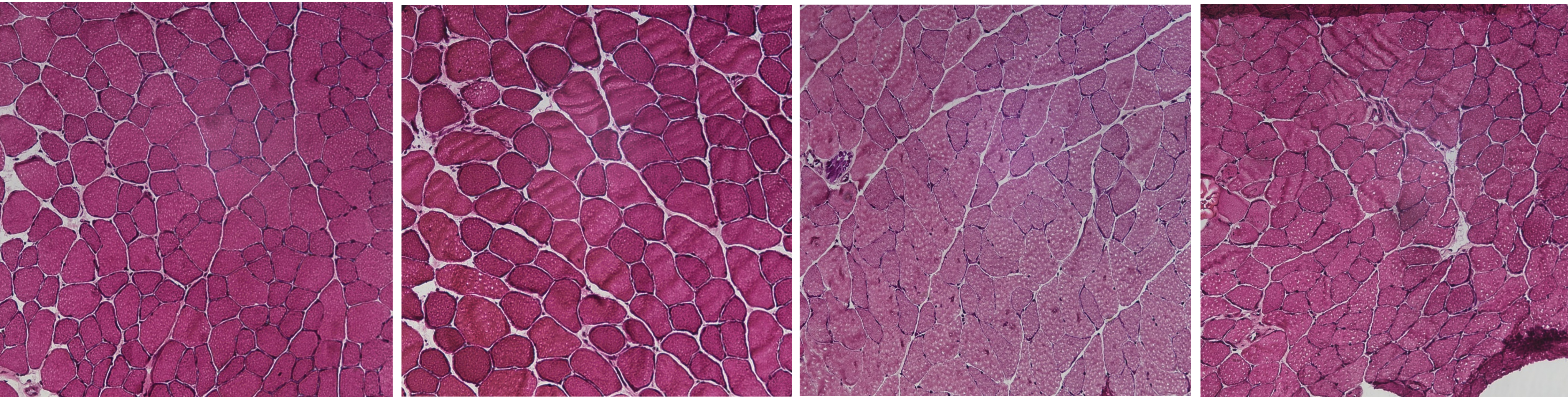

Nikon ECLIPSE Ni

20X magnification

GAPDH

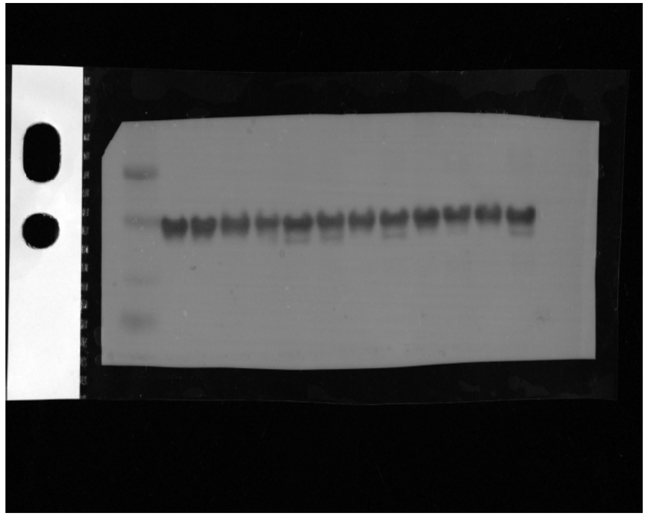

IGF-1

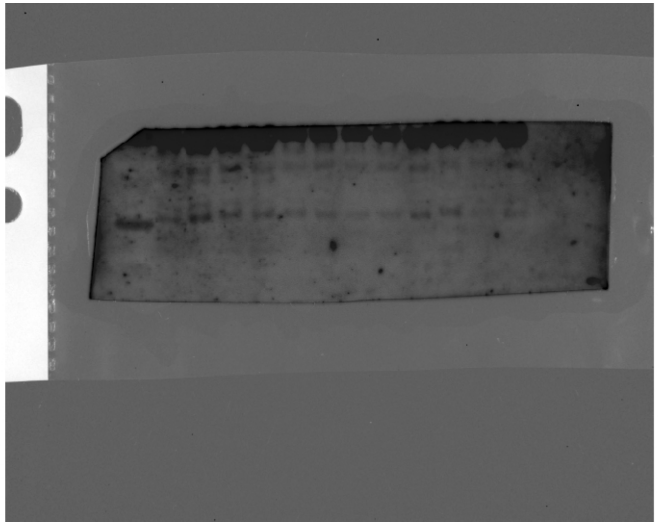

mTOR

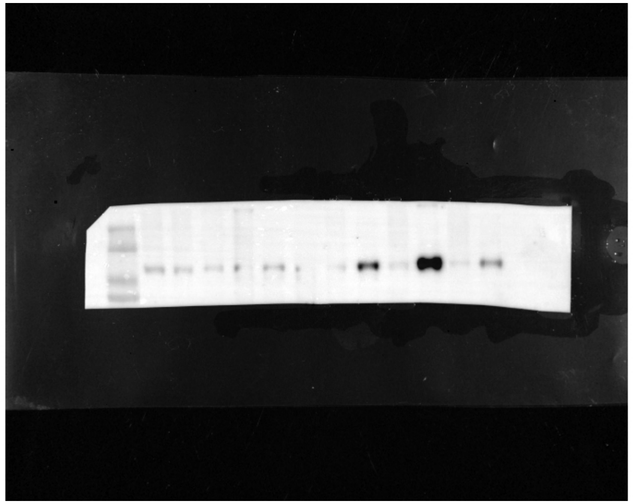

S6K1

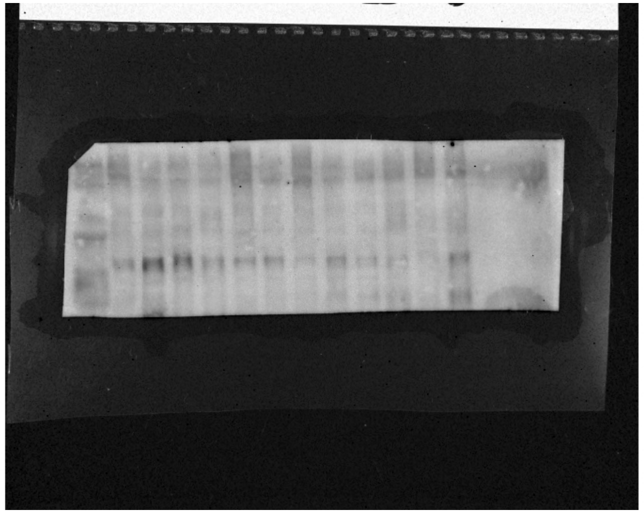

From the front, each three lanes were loaded in the order of Young-CON, Young-EXE, Old-CON and Old-EXE, respectively.

B-Actin

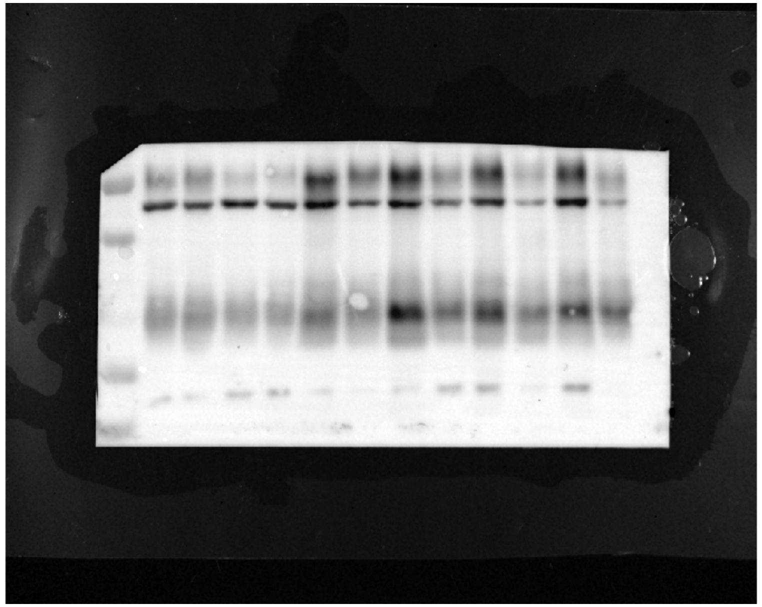

VEGF

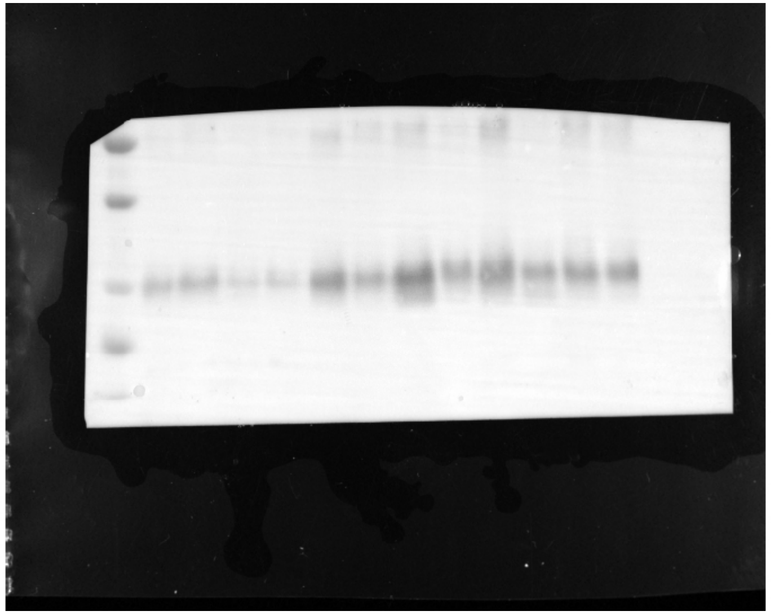

VEGFR2

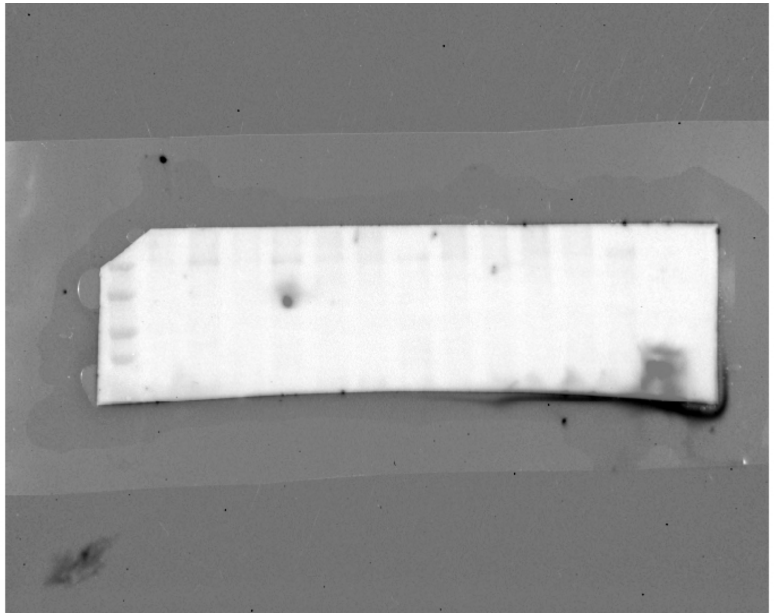

From the front, each three lanes were loaded in the order of Young-CON, Young-EXE, Old-CON and Old-EXE, respectively.

Young-CON **Young-EXE** **Old-CON** **Old-EXE**

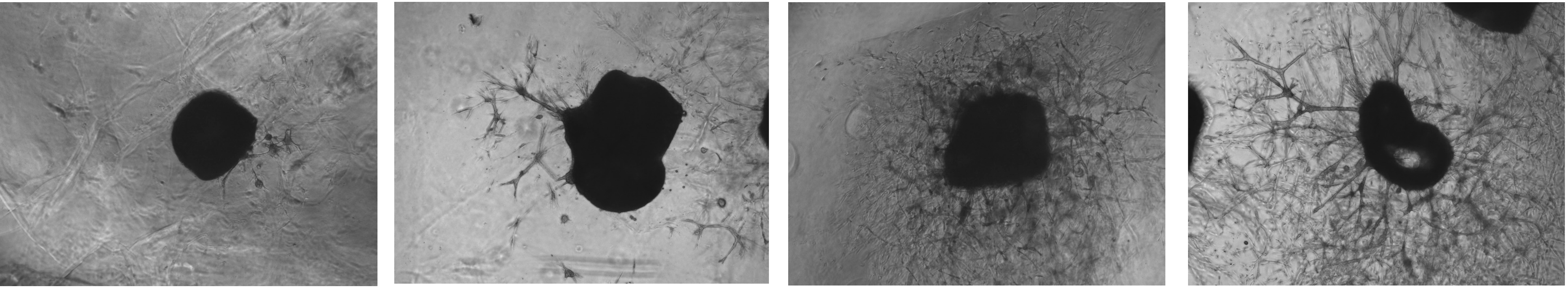

Mouse aorta
EVOS M5000
10X magnification

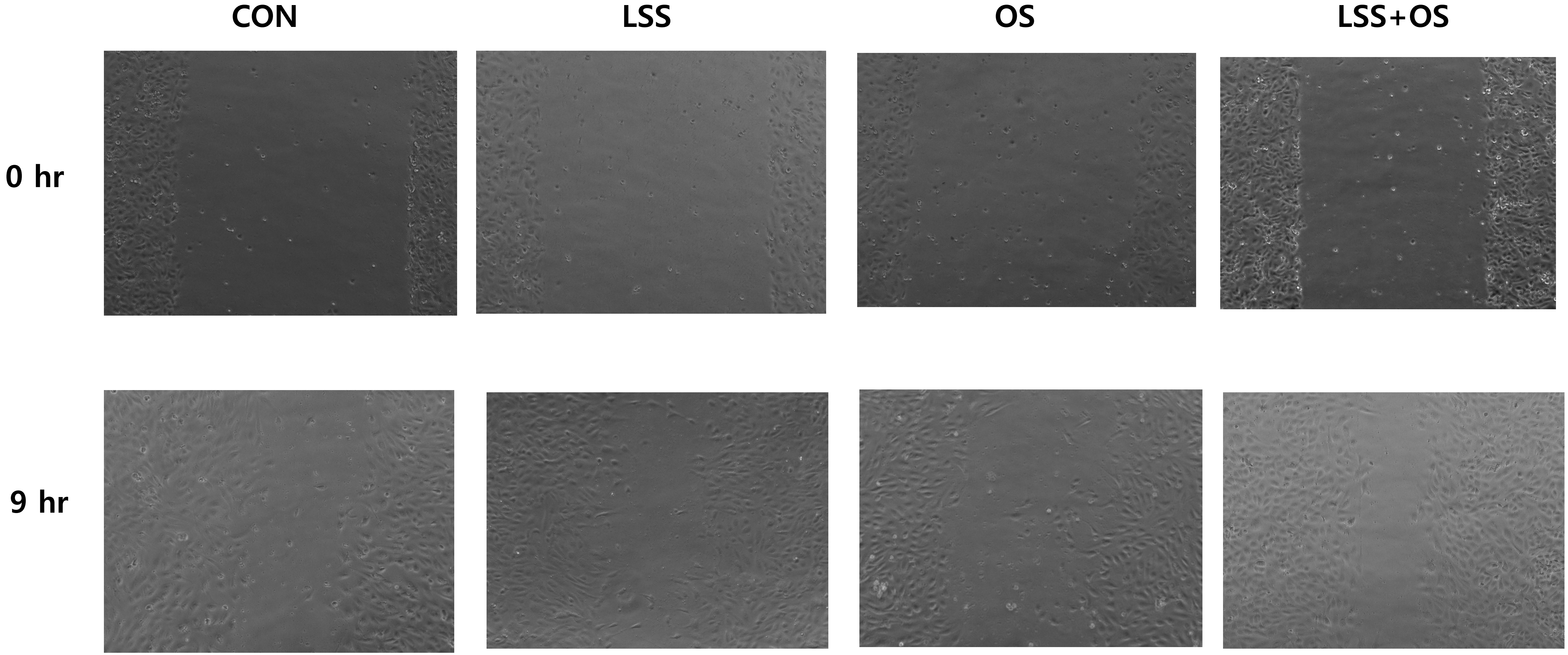

HUVEC (Human umbilical vein endothelial cells)
EVOS M5000
10X magnification

CON LSS OS LSS+OS

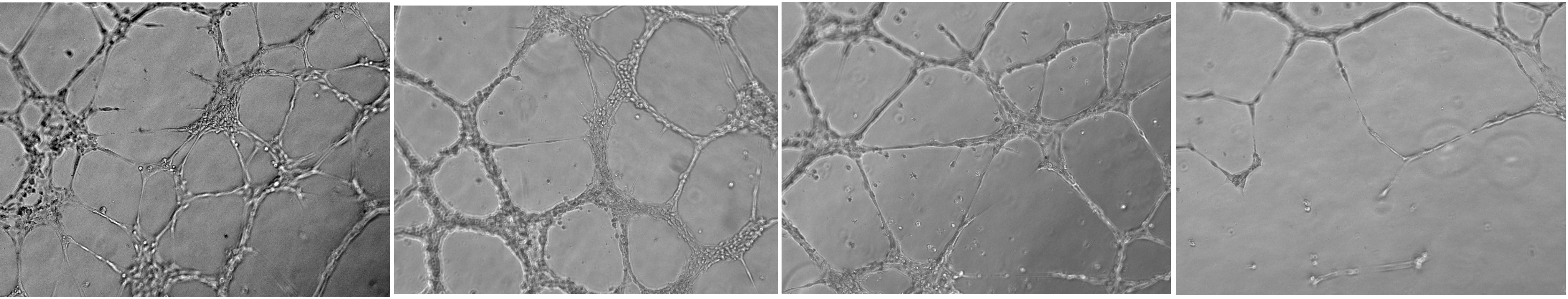

HUVEC (Human umbilical vein endothelial cells)
EVOS M5000
10X magnification
